# THE SHORT- AND LONG-TERM IMPACTS OF DEMENTIA ON PREVENTIVE CARE UTILIZATION AND HEALTH BEHAVIORS

**DOI:** 10.1093/geroni/igac059.1616

**Published:** 2022-12-20

**Authors:** Zhuoer Lin, Xi Chen

**Affiliations:** Yale University, New Haven, Connecticut, United States; Yale University, New Haven, Connecticut, United States

## Abstract

Identifying early and consistent signals of dementia risk may offer people more time to prepare for the future, helping to delay the onset or slow the progression of dementia. Using the 1995-2018 waves of Health and Retirement Study, we offered novel evidence on the impacts of dementia and its precursors on a rich set of preventive care utilization and health behaviors in a longitudinal sample of Americans aged 50 and older. Leveraging both within- and between-individual variations in an event study, we characterized short- and long-term dynamic changes in preventive care and health behaviors relative to the incidence of dementia, suggesting early behavioral indicators of the disorder. 3,488 persons with incidence of dementia were identified and 22,619 consecutive person-waves around the incidence of dementia were included to estimate short-term effect. Individuals who never developed dementia were further included as the comparison group for long-term estimation. Results from statistical models showed that incidental dementia was associated with significant reductions in preventive care utilization, including cholesterol test, flu shot, mammogram, prostate test, and dental visit. Significant declines were also found in physical activities and body weights. Moreover, growing declines were observed in preventive care utilization, physical activities, and body weight 4-6 years prior to the incidence of dementia, with escalating impact over time. These findings highlight vulnerability and costs dementia risk may accrue to disrupt preventive care utilization and health behaviors. Targeted interventions are needed to detect signals associated with dementia risk to shield individuals from adverse behavioral and health consequences.

